# Validation of the eyeTelemed IOPvet indentation tonometer for use in dogs

**DOI:** 10.1111/vop.13215

**Published:** 2024-04-02

**Authors:** Lydia E. Kapeller, Ava G. Cabble, Phillip N. Buckman, Christine D. Harman, Amanda L. Jacobson, Frank R. Lawrence, András M. Komáromy

**Affiliations:** ^1^ Department of Small Animal Clinical Sciences College of Veterinary Medicine, Michigan State University East Lansing Michigan USA; ^2^ Center for Statistical Training and Consulting Michigan State University East Lansing Michigan USA

**Keywords:** *ADAMTS10*‐open‐angle glaucoma, canine glaucoma, direct manometry, glaucoma screening, intraocular pressure, tonometry

## Abstract

**Objective:**

To assess the accuracy of canine intraocular pressure (IOP) estimates from the eyeTelemed IOPvet indentation tonometer.

**Animals Studied:**

Part 1 included 54 eyes from 28 Beagle dogs—23 *ADAMTS10*‐mutants with open‐angle glaucoma and 5 normals. Part 2 involved five normal canine ex vivo globes.

**Procedure:**

Part 1 (in vivo) compared IOPvet estimates in normal and glaucomatous dogs to Reichert Tono‐Vera® Vet rebound tonometry. The three IOPvet estimates were green (normal; <20 mmHg, according to the manufacturer), yellow (elevated; 20–30 mmHg), and red (high; >30 mmHg). In Part 2 (ex vivo), the pressure inside freshly enucleated normal canine eyes was progressively increased from 5 to 80 mmHg and compared to IOPvet estimates. Descriptive statistics compared IOPvet estimates to rebound tonometry and direct manometry, with the threshold from normal to glaucoma set at 30 mmHg.

**Results:**

In Part 1 (in vivo), normal pressures (≤30 mmHg) were mainly identified correctly as green or yellow—110 of 111 estimates, corresponding to a specificity of 99%. Only 16 of 125 affected estimates were correctly displayed in the >30‐mmHg range; the remaining 109 showed ≤30 mmHg, corresponding to a sensitivity of 13%. In Part 2 (ex vivo), all normal pressures were correctly estimated with green, but 64 of 88 manometric IOPs >30 mmHg were falsely estimated as 20–30 mmHg.

**Conclusions:**

The IOPvet is inaccurate in estimating canine IOP with a low sensitivity at identifying dogs with IOP > 30 mmHg. Canine‐specific instrument revision is required to correctly identify elevated (yellow = 20–30 mmHg) and high (red >30 mmHg) IOPs.

## 1 INTRODUCTION

Glaucoma is a leading cause of irreversible blindness in dogs, with intraocular pressure (IOP)‐associated biomechanical stress being the main identifiable cause of optic nerve degeneration.^1^ The measurement of IOP by tonometry is essential to glaucoma diagnostics and therapy assessment. A major challenge is that during office visits with veterinary ophthalmologists, tonometry only provides snapshots, and dangerous IOP spikes between office visits remain undiagnosed.^2^ While an increasing number of primary care and emergency veterinarians invest in applanation and rebound tonometers, this purchase is too costly for many. Much improvement in canine glaucoma diagnostics and management could be achieved by broader access to affordable tonometry, either through primary care veterinarians or home monitoring by dog owners.^2^

**FIGURE 1:**
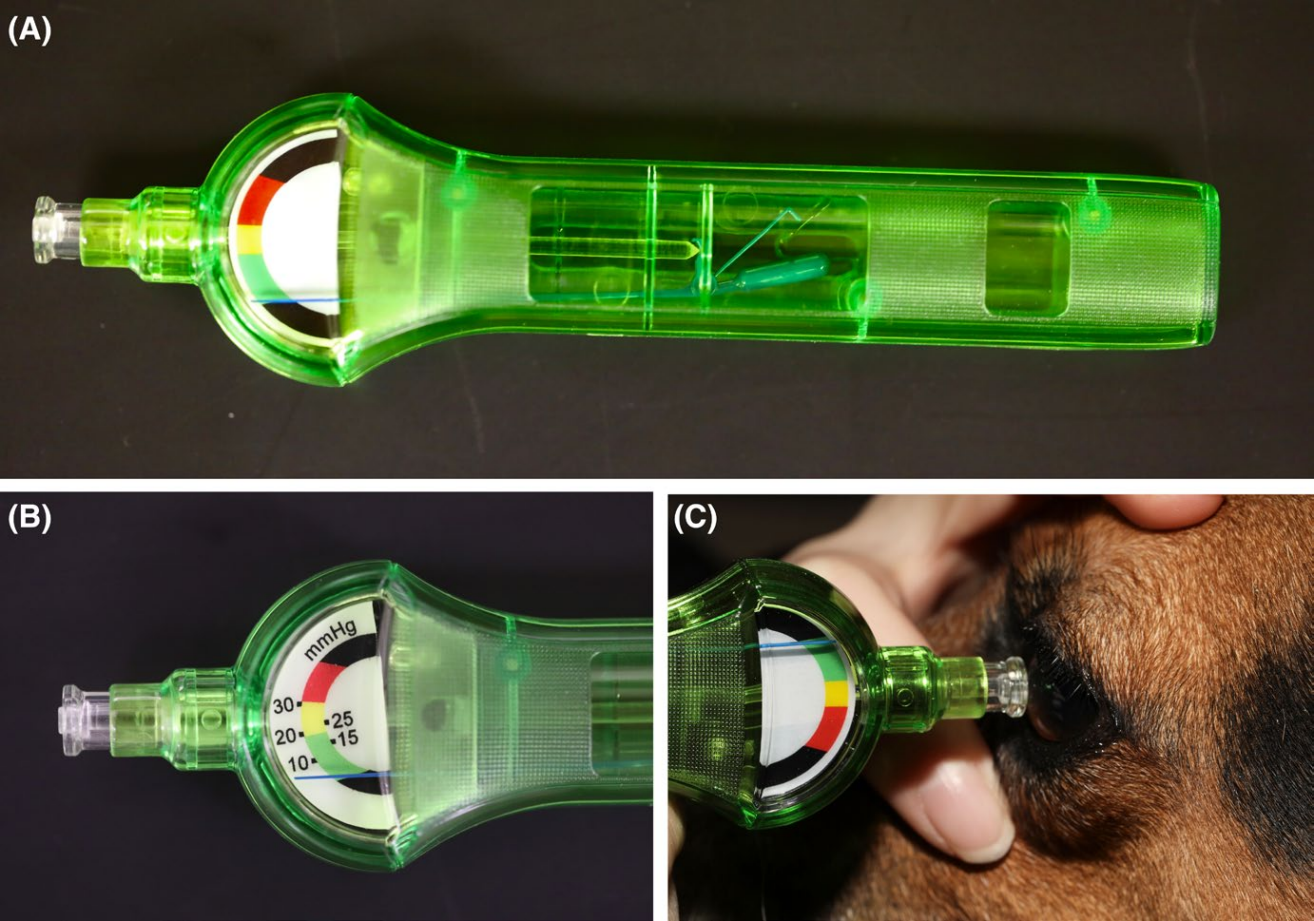
The eyeTelemed IOPvet indentation tonometer: (A) with color scale only, (B) with color and number scale, and (C) application on a dog.

The eyeTelemed IOPvet indentation tonometer (Ingeneus Pty. Ltd) is a new‐to‐the‐market, low‐cost, single‐use device that is being advertised as a low‐cost option to estimate IOPs in veterinary species (Figure 1). The device provides three IOP ranges that are color coded as green or normal (<20 mmHg), yellow or elevated (20–30 mmHg), and red or high (>30 mmHg).^3^ As there is no published report on the use of this device in animals, we assume that the device is identical to the Ingeneus eyePressure© device designed for use in humans.^4^ This study aimed to evaluate the use of the IOPvet indentation tonometer in purpose‐bred normal Beagle‐derived dogs and dogs with *ADAMTS10*‐open‐angle glaucoma (*ADAMTS10*‐OAG). In addition, the device was validated by comparison to direct manometric IOPs in normal ex vivo canine eyes.

## 2 METHODS

### 2.1 Study design and animals

The study consisted of two parts: In Part 1 (in vivo), IOPvet estimates from purpose‐bred normal and glaucomatous Beagle‐derived dogs were compared with IOPs obtained with the previously evaluated Reichert Tono‐Vera® Vet rebound tonometer.^5^, ^6^ In Part 2 (ex vivo), IOPvet estimates were performed at defined set pressures in freshly enucleated eyes and compared to direct manometric IOPs.

**FIGURE 2:**
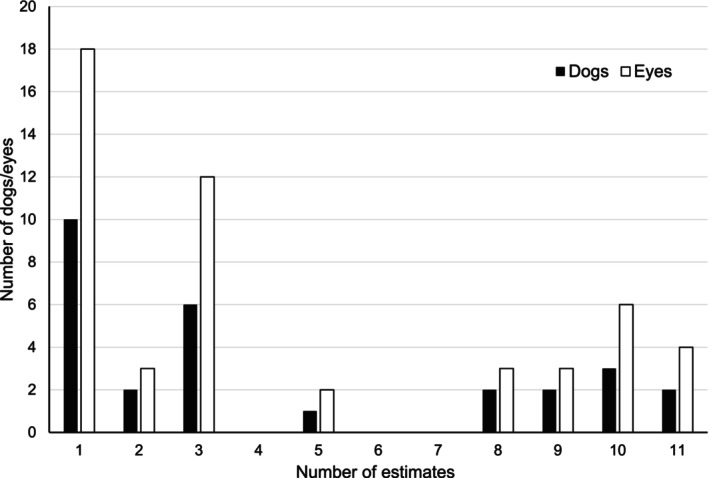
Distribution of dogs and eyes per number of estimates. While most dogs and eyes were only tested once, some were assessed multiple times on different days and times relative to IOP‐lowering medications.

For Part 1 (in vivo), 265 estimates were performed on 55 eyes from 28 purpose‐bred Beagle‐derived dogs. Of these dogs, 23 were at different stages of OAG caused by the G661R missense mutation in the *ADAMTS10* gene (*ADAMTS10‐*OAG).^7^ Five dogs were unaffected by glaucoma; two of them were carriers of the *ADAMTS10* missense mutation, and three were wild types concerning the *ADAMTS10* mutation. The median age of the dogs was 4.2 years (range: 1.0–7.1 years). Of the 28 enrolled dogs, 15 were females, 12 were males, and one was a neutered male. Some of the dogs and eyes were used multiple times (Figure 2) because they were on IOP‐lowering treatment for advanced OAG, latanoprost 0.005% (Bausch & Lomb Incorporated) and/or dorzolamide HCl/timolol maleate 2%/0.5% (Bausch & Lomb Incorporated) ophthalmic solutions. In addition, some of the same dogs were treated once with atropine sulfate 1% ophthalmic solution (Bausch & Lomb Incorporated) for iridocycloplegia, resulting in a more considerable IOP increase. These dogs were assessed before and at various times after IOP‐modifying eye drop administration to generate a broader range of pressure readings and, therefore, a more comprehensive comparison between the tonometry methods. The atropine treatment was reversed with latanoprost 0.005% ophthalmic solution following tonometry. In addition, dogs with anticipated or confirmed IOP >40 mmHg were treated with gabapentin (10–20 mg/kg PO every 8–12 h; ACI Healthcare USA, Inc.) to mitigate potential discomfort from severely elevated IOP. All data points were collected in the same room but on various days and times. All the dogs ate the same diet. They were group‐housed in the same environment at (the Michigan State University College of Veterinary Medicine Vivarium) with a 12‐h light and 12‐h dark cycle.

For Part 2 (ex vivo), five freshly enucleated normal left eyes from five male mongrel dogs were used. The eyes were enucleated postmortem from dogs euthanized as part of an unrelated study, shipped overnight on ice by a contract laboratory, and used within 24 h of collection. No dogs were euthanized solely for this study. The cadaver eyes were confirmed to be free of lesions that could affect the study by a board‐certified veterinary ophthalmologist (AMK) using slit‐lamp biomicroscopy (Kowa SL17; Kowa Company) and binocular indirect ophthalmoscopy (Keeler All Pupil II; Keeler Instruments), and condensing lens (Double Aspheric Pan Retinal 2.2D; Volk Optical).

### 2.2 Part 1: In vivo tonometry

The protocol was the same for each dog and eye: IOPs were first assessed in mmHg with the Reichert Tono‐Vera® Vet tonometer (Reichert Technologies) using the “dog” setting recently evaluated in this species.^5^, ^6^ The dogs were lightly restrained on an examination table, with vigilance not to put manual pressure on the globe. Using the ActiView™ positioning system, three readings were averaged automatically when the tonometer was positioned correctly over the central cornea. The device added additional readings (up to six total) if any of the three initial readings deviated more than 10% from the median value. A green ring around the averaged IOP indicated that the final number displayed an average of three readings that fell within this 10% tolerance. Tonometry was repeated if, after six readings, the built‐in screen indicated that this requirement was not met, that is, fewer than three readings were within 10% of the median. These confidence results were based on the accuracy specifications provided by the manufacturer, which are ±1.2 mmHg for IOPs between 5 and 19 mmHg and ± 2.2 mmHg for IOPs between 20 and 60 mmHg.^8^

The Tono‐Vera® Vet tonometry was performed by Investigator 1 and concealed from Investigator 2. Subsequently, proparacaine HCl 0.5% ophthalmic solution (Alcon Laboratories, Inc.) was administered for ocular surface anesthesia. Investigator 2 then estimated the IOP with the eyeTelemed IOPvet by recording the color of the device's color wheel—green, yellow, or red (Figure 1). Each estimate was performed two to three times to ensure consistency of the result. IOPvet IOP estimates falling between two colors were recorded as green/yellow or yellow/red—such IOP estimates were to be noted and plotted but ultimately excluded from statistical analysis. The two investigators' roles of estimating pressures with either the Tono‐Vera® Vet or the eyeTelemed IOPvet were alternated between each dog. A new IOPvet device was used for each procedure day.

### 2.3 Part 2: Ex vivo tonometry

The study design followed previously published protocols.^9^, ^10^, ^11^ Eyes were positioned so that the corneal surface was perpendicular to the horizontal surface placed upon and secured in place. The anterior chamber was cannulated through the corneal limbus using two 25‐gauge 5/8‐inch needles, one at the 3 o'clock position, which was attached to a 1‐L bag of Plasma‐Lyte A injection fluid (PLASMA‐LYTE A Injection pH 7.4, Baxter Healthcare Corporation), and one at the 9 o'clock position which was attached to a calibrated manometer (Traceable® Manometer Model 3460; Control Company). No leakage was observed at any cannulation site. The manometric IOP was increased in 5‐mmHg increments from 5 to 40 mmHg and then in 10‐mmHg increments to 80 mmHg by raising the fluid bag. The corneal surface was irrigated with sterile balanced salt solution (BSS® Sterile Irrigation Solution, Alcon Laboratories, Inc.) at each IOP level before performing tonometry.

Estimates were taken by the same unmasked investigator (LEK) on all five eyes with the eyeTelemed IOPvet at each setpoint (5, 10, 15, 20, 25, 30, 35, 40, 50, 60, 70, and 80 mmHg) starting at 5 mmHg, and slowly raising the fluid bag until reaching 80 mmHg. Manometric setpoints ±1 mmHg were accepted and accurately recorded. Following the same procedure as in Part 1, the IOPs were estimated with the IOPvet by recording the color of the device's color wheel—green, yellow, or red. IOPvet IOP estimates falling between two colors were recorded as green/yellow or yellow/red but excluded from statistical analysis. For each setpoint and eye, the IOPvet IOP estimate was performed two to three times to ensure consistency of the result. A new IOPvet device was used for each eye.

### 2.4 Statistics

Descriptive statistics compared the IOPvet estimates to Tono‐Vera® Vet rebound tonometry IOPs (Part 1) and direct manometry (Part 2) under the following consideration: Given the IOPvet manufacturer's option to differentiate between green and yellow or yellow and red, we decided that the 30‐mmHg threshold was more critical than the 20‐mmHg threshold to distinguish between glaucoma‐unaffected and affected canine eyes that may require therapy. In Part 1 (in vivo), sensitivity, specificity, and positive and negative predictive values were calculated.

## 3 RESULTS

### 3.1 IOPvet usage

The IOPvet tonometer was easy to use for non‐veterinarians to collect IOP estimates. There was generally good agreement between the two to three estimates on the same eye at any specific time or IOP setpoint. The canine subjects generally tolerated the device well when the anesthetized corneal surface was touched by its foot plate and plunger (Figure 1C).

### 3.2 Part 1: In vivo tonometry

**FIGURE 3:**
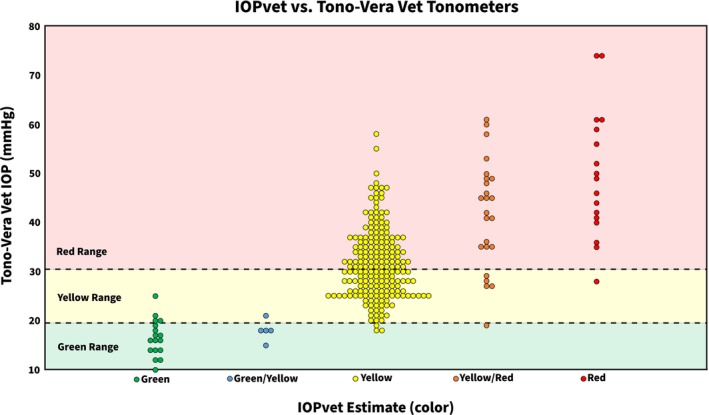
In vivo comparison of IOPvet estimates (horizontal axis) with Tono‐Vera® Vet IOPs (vertical axis). If the estimates were correct, the green, yellow, and red shaded areas indicate where the data points should be located. Additional green/yellow and yellow/red categories were added for in‐between estimates but were not used in the statistical analysis.

A total of 265 coupled IOP estimates were taken from the 54 eyes of 28 dogs with both the Tono‐Vera® Vet and IOPvet tonometers. The Tono‐Vera® Vet pressures were considered the actual IOP for this part of the study.^6^ The color‐coded IOPvet estimates were plotted relative to the Tono‐Vera® Vet IOPs (Figure 3), and the results are summarized in Table 1. Of the 265 estimates, 29 consistently fell between two color categories (green/yellow or yellow/red) and were not included in descriptive statistics.

**TABLE 1:** Part 1 (in vivo): Number of IOPvet estimates compared to Tono‐Vera® Vet IOPs.

	IOPvet estimates		
	Green (<20 mmHg)	Yellow (20–30 mmHg)	Red (>30 mmHg)
Tono‐Vera® Vet IOP (mmHg)			
<20	13	3	0
20–30	4	90	1
>30	0	109	16

With one exception (red for 28 mmHg) all other normal IOPs ≤30 mmHg (*n* = 110) were correctly estimated as green or yellow. Of all the 125 glaucomatous eyes (IOP >30 mmHg), only 16 were diagnosed correctly with red, while 109 estimates were falsely diagnosed as normal or yellow. If the collection of our estimates was considered a population, which is not accurate due to repeat assessments, the sensitivity and specificity of the IOPvet estimates would be 13% and 99%, respectively (Table 2). The positive and negative predictive values would be 94% and 50%, respectively (Table 2). Of all the green estimates (*n* = 17), 13 were < 20 mmHg, while four were false with IOP ≥20 mmHg. Of all the yellow estimates (*n* = 202), only 90 were in the 20–30 mmHg range, while 3 were <20 mmHg and 109 were >30 mmHg. The overall yellow range was 18–58 mmHg.

**TABLE 2:** Part 1 (in vivo): Sensitivity, specificity, and predictive values with 30 mmHg threshold.

Glaucoma (> 30 mmHg)	Normal (≤30 mmHg)	Predictive values
True affected: 16	False affected: 1	Positive: 94%
False normal: 109	True normal: 110	Negative: 50%
Sensitivity: 13%	Specificity: 99%	

### 3.3 Part 2: Ex vivo tonometry

**FIGURE 4:**
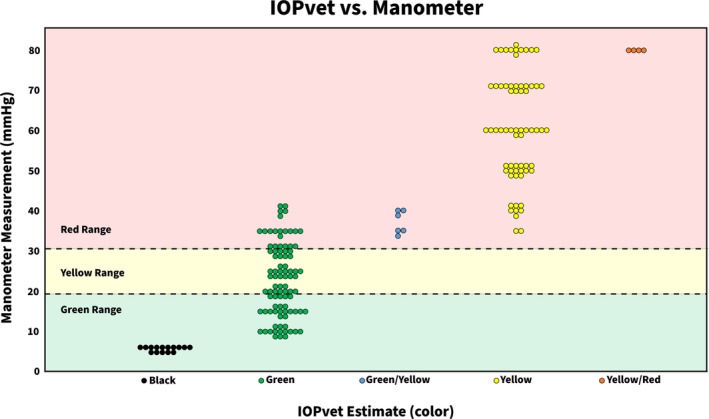
Ex vivo comparison of IOPvet estimates (horizontal axis) with direct manometric IOP measurements (vertical axis) from 5 to 80 mmHg. If the estimates were correct, the green, yellow, and red shaded areas indicate where the data points should be located. Additional categories of black (very low pressure) as well as green/yellow and yellow/red (in‐between estimates) were added but were not used in statistical analysis.

The results of the ex vivo comparison between IOPvet estimates and direct manometry in five canine globes are summarized in Figure 4 and Table 3. All estimates at the 5‐mmHg setpoint remained in the black range, located below the green range. Of all the black and green estimates (*n* = 105) only 53 were indeed <20 mmHg, while the remaining green estimates corresponded to manometric pressures of 20–30 mmHg (*n* = 28) and > 30 mmHg (*n* = 24), respectively. All the normal manometric pressures <20 mmHg were correctly estimated as green. None of the yellow estimates were in the predicted range of 20–30 mmHg, but all were >30 mmHg (*n* = 64), precisely 35–80 mmHg. None of the estimates were clearly in the red, but four estimates at 80 mmHg were located at the yellow/red transition.

**TABLE 3:** Part 2 (ex vivo): Number of IOPvet estimates compared to direct manometric pressures measured from 5 to 80 mmHg.

	IOPvet estimates		
	Black or green (<20 mmHg)	Yellow (20–30 mmHg)	Red (>30 mmHg)
Direct manometric IOP (mmHg)			
<20	53		
20–30	28		
>30	24	64	

## 4 DISCUSSION

Testing an extensive range of IOPs in normal and glaucomatous dogs and ex vivo normal canine globes revealed that the eyeTelemed IOPvet indentation tonometer is unreliable in accurately estimating IOPs in this species. In Part 1 of our study, most green and red estimates were correct when examining normal and glaucomatous dogs. According to the manufacturer, these estimates correspond to <20 mmHg or normal (green) and >30 mmHg or high (red), respectively.^3^ Most of our in vivo estimates were in the yellow range, defined by the manufacturer as 20–30 mmHg or elevated,^3^ but covering an actual range of 18–58 mmHg from normal to severely elevated IOPs. This means that yellow estimates are unreliable. The safest assumption in a clinical application would be to consider all yellow estimates elevated and an indication for IOP‐lowering therapy and/or for a referral to a veterinary ophthalmologist. With this assumption, no harm will be done by missing glaucomatous dogs, but many normal eyes will be diagnosed falsely with glaucoma.

Our results from Part 2 on ex vivo normal canine globes also suggest that all yellow and red IOPvet estimates should be considered likely glaucoma diagnoses. We obtained yellow estimates for IOPs in the 40–80‐mmHg range, contrasting the manufacturers' suggested 20–30 mmHg; we did not get precise red estimates even at 80 mmHg, where yellow and yellow/red estimates were obtained instead. According to the manufacturer, any IOP >30 mmHg should be in the red range—this was not the case in our ex vivo experiment. An important finding in Part 2 was the extensive range of IOPs (10–40 mmHg) that were estimated as green or normal (<20 mmHg), according to the manufacturer.^3^ Based on these findings, a green estimate in a dog eye is entirely unreliable. Interestingly, the black estimate, which is not described by the manufacturer, reliably corresponded to a low pressure of 5 mmHg.

When summarizing both parts of our study, we conclude that a canine glaucoma diagnosis will likely not be missed if all yellow and red estimates are referred to a veterinary ophthalmologist for further workup or started on IOP‐lowering medication. However, applying this recommendation will falsely diagnose many dogs with elevated IOPs. Any green estimates cannot be trusted as the IOPs could be normal or moderately to severely elevated.

The availability of a low‐cost tonometer would be beneficial to make reliable glaucoma diagnosis and monitoring more widely available to dogs.^2^ Because the IOPvet indentation tonometer requires ocular surface anesthesia, it is not designed for home monitoring of IOPs of dogs at risk by their owners. However, if canine‐specific validation data is taken into account, the IOPvet could be helpful for primary care and emergency veterinarians and veterinary medical personnel for whom the investment in a more expensive electronic applanation or rebound tonometer is financially not feasible.

The IOPvet is an indentation tonometer similar to the Schiøtz tonometer.^12^, ^13^, ^14^ With indentation tonometry, the degree of corneal indentation by a defined force provides IOP measurements.^12^, ^13^, ^15^ With the Schiøtz tonometer, a weighted metal plunger exposed to gravity provides this defined force on the cornea.^12^ The Schiøtz tonometer requires the corneal surface to be in a horizontal plane for gravity to work. The required head positioning is not always easily achieved and/or tolerated by the animal.^14^ Because of its internal spring mechanism, gravity is unnecessary when using the IOPvet, making it more user‐ and animal‐friendly than the Schiøtz tonometer as IOPs can be estimated with the animal in an upright position (sitting or standing).

The Schiøtz indentation tonometer was extensively validated for several animal species, including the dog, with establishments of human and canine tables for conversion of Schiøtz scale readings to IOPs.^14^, ^16^, ^17^, ^18^, ^19^ In contrast, no published validation data exist for using the IOPvet in dogs or any other veterinary species. We suspect that the IOPvet was derived from or is identical to the Ingeneus eyePressure© device marketed for use in humans.^4^ Based on our data, we further suspect that the IOPvet is calibrated for the human rather than the canine eye.

The discrepancies between many of our IOPvet indentation tonometer estimates and the true canine IOP can be explained by the extensive historical data available for the Schiøtz tonometer.^14^, ^16^, ^17^, ^18^, ^19^ Differences between the canine and human corneal indentation and Schiøtz scale readings are based on species differences in corneal curvature, thickness, and ocular rigidity and elasticity.^12^, ^13^, ^19^, ^20^ We assume that these differences between species were not considered by the IOPvet manufacturer and that the instrument may be calibrated for the human eye. The IOPvet is marketed internationally for animal use without transparency regarding the likely missing species‐specific validation.

Our study has several weaknesses, which should not affect the general conclusions. While the number of in vivo measurements and estimates were sizeable, several dogs and eyes were measured multiple times on different days and at various intervals since the last IOP‐altering eye drop administration. The larger numbers allowed us to perform more detailed analyses, but calculated sensitivity, specificity, and positive and negative predictive values are estimates that cannot be translated to the larger canine population. The *ADAMTS10* missense mutation in the OAG‐affected eyes affects the eye's biomechanics, such as a softer sclera and possibly cornea, which could result in an IOP underestimation of indentation tonometry.^21^, ^22^, ^23^ Considering the large number of published data on canine *ADAMTS10*‐OAG and tonometry, including Schiotz tonometer validation,^19^ we do not think that the effect of the mutation is a major consideration when interpreting our results. To compensate for any shortcomings in the in vivo Part 1 of the study, we performed the ex vivo Part 2 on normal globes of young adult dogs. The combined results of both study parts should be sufficiently robust to draw clinically relevant conclusions. If an improved instrument for veterinary application becomes available in the future, the selection of dogs and eyes can be improved and expanded for its validation. Other animal species still need to be tested with the IOPvet.

In conclusion, while the eyeTelemed IOPvet indentation tonometer represents a cost‐effective, easy‐to‐use, and well‐tolerated method that makes tonometry accessible to a broader field of primary care veterinarians and their animal patients, our in vivo and ex vivo canine data show that the IOPvet pressure estimates are not reliable and have to be interpreted very carefully when obtained in dogs. Most importantly, the IOPvet is inaccurate in estimating canine IOP with a low sensitivity of 13% at identifying dogs with IOP > 30 mmHg.

## AUTHOR CONTRIBUTIONS

**Lydia E. Kapeller:** Conceptualization; data curation; formal analysis; investigation; methodology; visualization; writing – original draft; writing – review and editing. **Ava G. Cabble:** Conceptualization; investigation; writing – review and editing. **Phillip N. Buckman:** Conceptualization; investigation; methodology; writing – review and editing. **Christine D. Harman:** Investigation; methodology; project administration; supervision; writing – review and editing. **Amanda L. Jacobson:** Investigation; methodology; writing – review and editing. **Frank R. Lawrence:** Formal analysis; methodology; validation; writing – review and editing. **András M. Komáromy:** Conceptualization; data curation; formal analysis; funding acquisition; methodology; project administration; resources; supervision; visualization; writing – original draft; writing – review and editing.

## CONFLICT OF INTEREST STATEMENT

AM Komáromy is a consultant for Reichert® Technologies and W. L. Gore & Associates, Inc. AM Komáromy received research funding from PolyActiva Pty. Ltd., CRISPR Therapeutics, and Advanced Ophthalmics LLC while the presented work was conducted. While AM Komáromy also serves as Editor‐in‐Chief of Veterinary Ophthalmology, he was not involved in the review of this manuscript. All other authors declare no conflict of interest.

## ETHICS STATEMENT

This study adhered to the Association for Research in Vision and Ophthalmology (ARVO) Statement for the Use of Animals in Ophthalmic and Vision Research and was approved by the Michigan State University Institutional Animal Care and Use Committee (IACUC). The Charles Rivers Laboratories IACUC approved the collection of donated tissues.

## Data Availability

The data that support the findings of this study are available from the corresponding author upon reasonable request.
